# Switching from Cation-Exchange Resin to Sodium Zirconium Cyclosilicate Hydrate in Patients on Hemodialysis

**DOI:** 10.3390/jcm15145406

**Published:** 2026-07-10

**Authors:** Naofumi Ikeda, Yuka Nodaira, Rie Kiyosumi, Kana Koinuma, Takanori Iwai, Kanako Nobe, Shiko Gen, Hiroo Kumagai

**Affiliations:** 1 Department of Nephrology, Sayama Renal Clinic, Sekishinkai Social Medical Foundation, Sayama 350-1305, Saitama, Japan; yuka-nodaira@saitama-sekishinkai.org (Y.N.); rie-kiyosumi@saitama-sekishinkai.org (R.K.); kana-koinuma@saitama-sekishinkai.org (K.K.); 2 Department of Nephrology, Saitama Sekishinkai Hospital, Sekishinkai Social Medical Foundation, Sayama 350-1305, Saitama, Japan; takanori-iwai@saitama-sekishinkai.org (T.I.); kanako-nobe@saitama-sekishinkai.org (K.N.); shikou-gen@saitama-sekishinkai.org (S.G.); hiroo-kumagai@saitama-sekishinkai.org (H.K.)

**Keywords:** hyperkalemia, magnesium concentration, hemodialysis, sodium zirconium cyclosilicate hydrate, cation-exchange resin, hypomagnesemia, vascular calcification

## Abstract

**Background/Objectives:** Hyperkalemia is a common complication in patients receiving maintenance hemodialysis (HD). Conventional cation-exchange resins, including sodium polystyrene sulfonate (SPS) and calcium polystyrene sulfonate (CPS), effectively lower serum potassium but may non-selectively bind magnesium. Sodium zirconium cyclosilicate (SZC) is a highly selective potassium binder with minimal affinity for magnesium. This study investigated changes in serum cation profiles and nutritional status after switching from conventional cation-exchange resins to SZC in patients undergoing maintenance HD. **Methods:** In this prospective, single-arm, before-and-after study conducted in routine clinical practice, 28 maintenance HD patients receiving SPS or CPS for chronic hyperkalemia were switched to SZC and followed for 12 weeks. Serum potassium, magnesium, corrected calcium, phosphorus, whole parathyroid hormone (whole-PTH), albumin, and the Geriatric Nutritional Risk Index (GNRI) were evaluated before and after the treatment transition. **Results:** Following the switch to SZC, median serum potassium increased modestly from 5.01 (4.66–5.39) to 5.26 (4.89–5.48) mEq/L (*p* = 0.031), whereas serum magnesium increased from 2.46 (2.29–2.66) to 2.66 (2.35–2.93) mg/dL (*p* < 0.001). Serum calcium, phosphorus, whole-PTH, albumin, and GNRI remained unchanged. Subgroup, correlation, and longitudinal analyses consistently supported the robustness of the observed increase in serum magnesium, irrespective of the previously prescribed resin or concomitant proton pump inhibitor use. **Conclusions:** Switching from conventional cation-exchange resins to SZC was associated with a significant increase in serum magnesium concentrations while maintaining acceptable potassium control and stable nutritional status in maintenance HD patients. These findings are consistent with the greater cation selectivity of SZC and suggest that replacement of conventional cation-exchange resins may contribute to preservation of serum magnesium concentrations. Further multicenter prospective studies evaluating magnesium metabolism together with clinically relevant outcomes are warranted to confirm these findings and clarify their clinical significance.

## 1. Introduction

Potassium is an essential electrolyte that is predominantly intracellular, with serum potassium levels maintained within a narrow physiological range under normal conditions [1]. Hyperkalemia—defined as serum potassium exceeding 5.0 mEq/L—is a frequent and potentially life-threatening complication in individuals with chronic kidney disease (CKD) and end-stage kidney disease (ESKD) [2,3,4]. In ESKD, impaired renal potassium excretion necessitates hemodialysis (HD) or peritoneal dialysis to regulate serum potassium levels. Despite dialysis, many patients experience persistent predialysis hyperkalemia, which has been independently associated with elevated all-cause and cardiac mortality [5,6]. Patients with ESKD on HD face high rates of hospitalization and mortality, particularly following extended dialysis intervals, with hyperkalemia as a potential major contributor [7,8]. A large retrospective analysis of 9347 US HD patients (the RE-UTILIZE study) demonstrated that predialysis hyperkalemia (K^+^ > 5.0 mEq/L) occurred in 74% of patients within 1 year and recurred in 60%, yet only 2.8% were prescribed an oral K^+^ binder, highlighting substantial undertreatment in this population [9].

Management of hyperkalemia in patients with ESKD on HD often requires pharmacological intervention in addition to dietary potassium restriction. Available treatment options include sodium polystyrene sulfonate (SPS), calcium polystyrene sulfonate (CPS), patiromer, and sodium zirconium cyclosilicate (SZC); however, patiromer (Veltassa^®^; approved in Japan in September 2024 and commercially available since March 2025) has recently become available in Japan. Among these agents, cation-exchange resins have been widely used for maintenance HD patients globally, with prescription rates of approximately 20% [10]. Despite their widespread use, cation-exchange resins are non-selective in their binding of cations and are associated with significant gastrointestinal side effects, including nausea, constipation, and in rare cases, colonic necrosis [11,12]. An important but often overlooked consequence of their non-selective binding is the co-excretion of other cations, including magnesium, alongside potassium in the gastrointestinal lumen.

SZC is a structurally distinct, highly selective potassium binder. Unlike conventional cation-exchange resins, SZC is not systemically absorbed and acts within the gastrointestinal lumen via a microporous zirconium silicate framework to selectively capture potassium and ammonium ions [13]. This mechanism promotes fecal potassium excretion and effectively lowers serum potassium levels [13,14,15]. Phase II and III clinical trials [14,15,16,17,18,19] have demonstrated SZC’s efficacy and safety in managing hyperkalemia in patients with CKD not undergoing dialysis, as well as those with heart failure and diabetes. Data from the DIALIZE trial further established SZC’s efficacy and tolerability in patients undergoing HD [20]. Real-world data from Singapore have also demonstrated SZC’s effectiveness in preventing hyperkalemia when dialysis is postponed or interrupted due to travel [21]. More recently, the DIALIZE-Outcomes trial—a large, international, randomized controlled trial—evaluated SZC’s effect on arrhythmia-related cardiovascular outcomes in patients on HD with recurrent hyperkalemia; although SZC did not significantly reduce the primary composite cardiovascular endpoint, it significantly improved normokalemia maintenance at 12 months [22].

Magnesium is an essential divalent cation involved in numerous physiological processes, including regulation of vascular smooth muscle function, inhibition of vascular calcification, and bone metabolism [23,24,25]. In patients receiving long-term HD, hypomagnesemia has been associated with increased cardiovascular mortality, progression of vascular calcification, and elevated fracture risk [26,27,28,29,30]. Despite these associations, the potential impact of cation-exchange resin-induced magnesium reduction on clinical outcomes in patients on HD has not been systematically investigated.

At our institution, we transitioned patients receiving maintenance HD from cation-exchange resins to SZC for hyperkalemia management. We comprehensively evaluated changes in serum potassium, magnesium, calcium, and phosphorus levels, alongside nutritional status, over a 12-week period before and after the switch. The primary aim of this study was to characterize the differential effects of SZC versus cation-exchange resins on serum cation profiles, with a particular focus on magnesium homeostasis.

## 2. Materials and Methods

### 2.1. Study Design

This was a prospective, single-arm, before-and-after study conducted in routine clinical practice. Maintenance hemodialysis (HD) patients with chronic hyperkalemia who provided written informed consent were switched from sodium polystyrene sulfonate (SPS) or calcium polystyrene sulfonate (CPS) to sodium zirconium cyclosilicate (SZC) and were prospectively followed for 12 weeks. Clinical, biochemical, and nutritional parameters were evaluated every two weeks throughout the study period.

The study protocol was approved by the Social Medical Corporation Foundation Sekishinkai Saitama Office Ethics Committee (Approval No. 2021-46) and was conducted in accordance with the principles of the Declaration of Helsinki. Written informed consent was obtained from all participants before enrollment. The study was registered with the University Hospital Medical Information Network Clinical Trials Registry (UMIN000052437).

### 2.2. Patients

Maintenance HD patients receiving SPS or CPS for the management of chronic hyperkalemia were eligible for enrollment. All patients had been treated with SPS or CPS for at least one year before switching (median duration, 5.5 years [IQR, 2.3–9.8 years]), and the prescribed dose had remained unchanged for at least seven months before enrollment.

Patients who provided written informed consent were switched to SZC and prospectively followed for 12 weeks. Patients who could not complete the study because of institutional infection-control measures requiring isolation dialysis, hospitalization, treatment modification, discontinuation of SZC, transfer to another facility, or withdrawal of consent during the observation period were excluded from the final analysis.

### 2.3. Treatment Protocol

Patients were switched from SPS or CPS to SZC. SZC was administered on non-dialysis days in accordance with the approved prescribing recommendations. Dose modification or treatment discontinuation was performed when clinically indicated.

Dietary counseling was provided by a registered dietitian before enrollment and every four weeks thereafter according to institutional practice. Dietary recommendations remained unchanged throughout the study period.

### 2.4. Laboratory Measurements

Blood samples were obtained immediately before the first HD session of each week (Monday or Tuesday, depending on the patient’s dialysis schedule) following the long interdialytic interval.

Serum potassium, magnesium, corrected calcium, phosphorus, whole parathyroid hormone (whole-PTH), albumin, and other routine biochemical parameters were measured at baseline and every two weeks during the 12-week observation period. Nutritional status was assessed using the Geriatric Nutritional Risk Index (GNRI).

Information regarding medications potentially affecting serum electrolyte and mineral metabolism, including renin–angiotensin system (RAS) inhibitors, proton pump inhibitors (PPIs), phosphate binders, active vitamin D preparations, calcimimetics, and magnesium-containing medications, was collected from the medical records. No clinically relevant changes in these medications occurred during the observation period, except for one patient who required dose adjustment of a RAS inhibitor and was therefore excluded from the final analysis. The dialysate magnesium concentration was fixed at 1.0 mEq/L throughout the study period.

### 2.5. Outcomes

The primary outcome was the change in serum magnesium concentration following the switch from SPS/CPS to SZC.

Secondary outcomes included changes in serum potassium, corrected calcium, phosphorus, whole parathyroid hormone (whole-PTH), nutritional status assessed by the Geriatric Nutritional Risk Index (GNRI), and other routine biochemical parameters.

### 2.6. Statistical Analysis

Continuous variables are presented as median (interquartile range). Comparisons between baseline and follow-up measurements were performed using the Wilcoxon signed-rank test. Correlations were evaluated using Spearman’s rank correlation coefficient. Longitudinal changes were analyzed as described in the Section 3. All statistical tests were two-sided, and a *p* value < 0.05 was considered statistically significant. Because of the exploratory nature of the study and the limited sample size, no adjustment for multiple comparisons was performed. Statistical analyses were performed using IBM SPSS Statistics version 24.0 (IBM Corp., Armonk, NY, USA).

## 3. Results

### 3.1. Patient Characteristics

Patient characteristics are summarized in Table 1. Twenty-eight patients completed the 12-week observation period and were included in the final analysis. The median age was 73.0 (interquartile range [IQR], 68–81) years, and the median duration of maintenance hemodialysis was 7.5 (IQR, 3.0–12.8) years. The most common primary kidney disease was diabetic nephropathy (*n* = 11), followed by chronic glomerulonephritis (*n* = 7), nephrosclerosis (*n* = 5), autosomal dominant polycystic kidney disease (*n* = 1), lupus nephritis (*n* = 1), and unknown etiology (*n* = 3). Before switching, 20 patients received sodium polystyrene sulfonate (SPS) and 8 received calcium polystyrene sulfonate (CPS). Following the switch, all patients initially received sodium zirconium cyclosilicate (SZC) at a fixed dose of 20 g/week (Table 1). The flow of patient enrollment is shown in Supplementary Figure S1.

### 3.2. Changes in Biochemical and Nutritional Parameters

Changes in biochemical and nutritional parameters before and after switching to SZC are summarized in Table 2.

Serum potassium increased significantly from 5.01 (4.66–5.39) to 5.26 (4.89–5.48) mEq/L (*p* = 0.031). Serum magnesium also increased significantly from 2.46 (2.29–2.66) to 2.66 (2.35–2.93) mg/dL (*p* < 0.001). No significant changes were observed in corrected calcium, inorganic phosphorus, whole parathyroid hormone (whole-PTH), or the Geriatric Nutritional Risk Index (GNRI).

### 3.3. Individual Changes in Serum Magnesium

**Figure 1:**
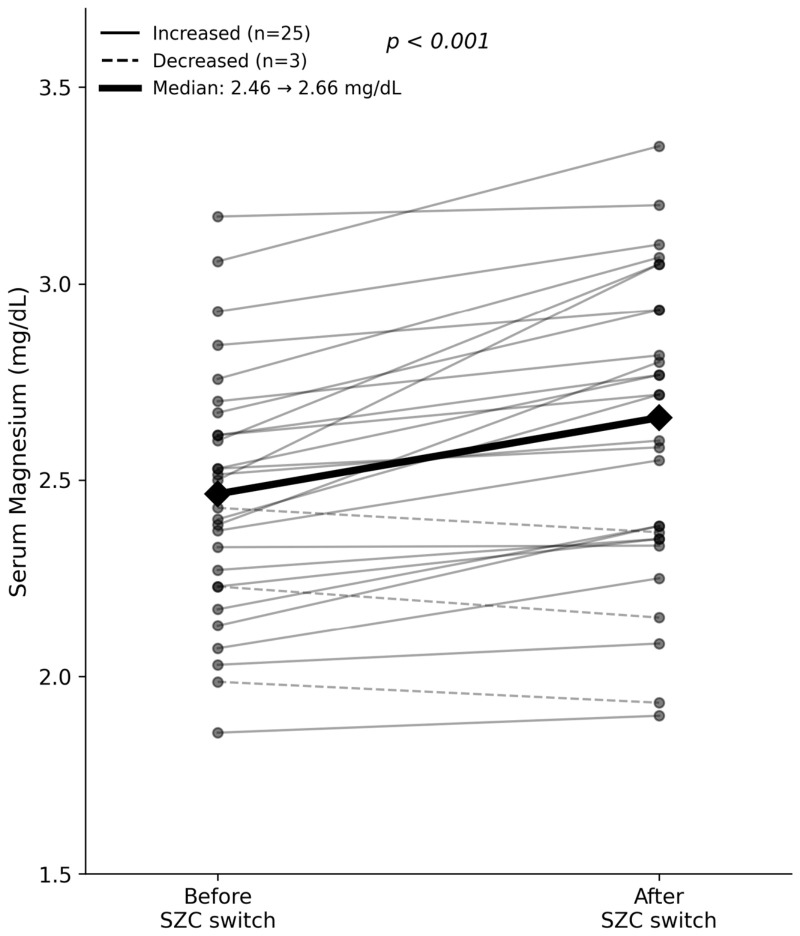
Individual changes in serum magnesium levels before and after switching from cation-exchange resin to sodium zirconium cyclosilicate in 28 hemodialysis patients. Each line represents one patient. Solid lines indicate patients with increased serum magnesium (*n* = 25); dashed lines indicate patients with decreased serum magnesium (*n* = 3). The bold line represents the median value (2.46 → 2.66 mg/dL). *p* < 0.001 by Wilcoxon signed-rank test.

Individual changes in serum magnesium concentrations are shown in Figure 1. Serum magnesium increased in 25 of the 28 patients (89%), whereas a decrease was observed in 3 patients (11%).

### 3.4. Subgroup Analysis According to Proton Pump Inhibitor Use

Fifteen patients were receiving proton pump inhibitors (PPIs), whereas 13 patients were not. The results of the subgroup analysis are summarized in Table 3.

Serum magnesium increased significantly following the switch to SZC in both the PPI group (2.27 [2.07–2.42] vs. 2.35 [2.15–2.80] mg/dL, *p* = 0.010) and the non-PPI group (2.61 [2.52–2.84] vs. 2.76 [2.66–3.08] mg/dL, *p* < 0.001). Serum potassium did not change significantly in either subgroup.

### 3.5. Subgroup Analysis According to the Previously Prescribed Resin

Subgroup analyses according to the previously prescribed potassium-binding resin are summarized in Table 4.

Serum magnesium increased significantly after switching to SZC in both the SPS group (2.41 [2.26–2.64] vs. 2.65 [2.36–2.96] mg/dL, *p* < 0.001) and the CPS group (2.52 [2.19–2.63] vs. 2.66 [2.23–2.81] mg/dL, *p* = 0.023). The magnitude of the increase in serum magnesium did not differ significantly between the two groups (*p* = 0.823).

Serum potassium increased significantly in the SPS group (*p* = 0.015) but not in the CPS group (*p* = 0.844).

### 3.6. Correlation Analysis

Spearman’s rank correlation analysis demonstrated no significant correlation between the previous dose of SPS/CPS and the change in serum magnesium concentration (ρ = 0.179, *p* = 0.373). Likewise, no significant correlation was observed between the changes in serum potassium and serum magnesium following the switch to SZC (ρ = 0.155, *p* = 0.430).

### 3.7. Longitudinal Changes in Serum Potassium and Magnesium

**Figure 2:**
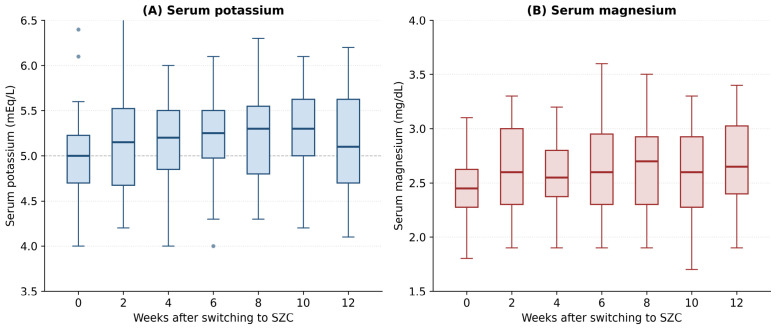
Box-and-whisker plots showing the longitudinal trajectories of serum potassium (**A**) and magnesium (**B**) over the 12 weeks following the switch from cation-exchange resin to SZC (*n* = 28 at each time point). Boxes represent the interquartile range (IQR) with the median indicated by the horizontal line; whiskers extend to the most extreme values within 1.5 × IQR from the box; dots indicate outliers.

Longitudinal changes in serum potassium and serum magnesium during the 12-week observation period are shown in Figure 2. Serum potassium remained stable throughout the study period, whereas serum magnesium increased after switching to SZC and was maintained during follow-up.

## 4. Discussion

In this prospective, single-arm, before-and-after study conducted in routine clinical practice, switching maintenance hemodialysis patients with chronic hyperkalemia from conventional cation-exchange resins (SPS or CPS) to sodium zirconium cyclosilicate (SZC) was associated with a significant increase in serum magnesium concentrations over the 12-week observation period. Although serum potassium increased modestly following the transition, concentrations remained within the recommended target range for maintenance hemodialysis patients. No clinically meaningful changes were observed in nutritional status, serum calcium, or phosphorus. Collectively, these findings suggest that replacement of conventional cation-exchange resins with SZC may influence magnesium homeostasis while preserving overall nutritional and mineral balance.

The increase in serum potassium deserves careful consideration. Previous comparative studies have reported that SZC provides potassium control comparable to that achieved with SPS or CPS in patients with CKD [31]. In contrast, serum potassium increased significantly after the transition in the present study. Because the SZC dose was fixed throughout the observation period whereas patients had previously received individualized doses of SPS or CPS, this finding most likely reflects differences in dose equivalence rather than reduced pharmacological efficacy of SZC. Furthermore, dietary adherence and medication compliance were not directly assessed and therefore cannot be excluded as additional contributors.

The increase in serum magnesium represents the principal finding of the present study. A pharmacological explanation is biologically plausible because conventional cation-exchange resins exchange potassium relatively non-selectively for sodium or calcium and are capable of binding other cations, including magnesium, whereas SZC exhibits substantially greater selectivity for potassium and ammonium ions [13]. Consequently, replacement of conventional cation-exchange resins with SZC may reduce unintended gastrointestinal magnesium binding and thereby contribute to preservation of circulating magnesium concentrations. However, because magnesium balance and intestinal magnesium handling were not directly evaluated, this mechanism should be regarded as a plausible hypothesis rather than a proven explanation.

Subgroup, correlation, and longitudinal analyses further supported this interpretation. Neither the previous dose of SPS/CPS nor the magnitude of the change in serum potassium was significantly associated with the increase in serum magnesium. Moreover, serum magnesium increased significantly after switching from both SPS and CPS, despite different potassium responses between these subgroups. Although these exploratory analyses do not establish causality, they suggest that the observed increase in serum magnesium cannot be explained solely by differences in potassium-binding potency or dose equivalence between conventional cation-exchange resins and SZC. Instead, the findings are consistent with the hypothesis that the greater cation selectivity of SZC contributes to preservation of serum magnesium concentrations.

The clinical relevance of magnesium homeostasis in patients receiving maintenance hemodialysis has been extensively investigated. Experimental studies have demonstrated that magnesium suppresses osteogenic transformation of vascular smooth muscle cells [23], whereas observational studies have consistently reported associations between lower serum magnesium concentrations and vascular calcification [24,25,32], cardiovascular events and mortality [26,27,33], and fracture risk [28,30]. These observations provide an important biological context for the present findings. However, our study evaluated only biochemical changes and was not designed to assess magnesium balance, vascular calcification, cardiovascular outcomes, bone metabolism, or fracture risk. Accordingly, the present findings should not be interpreted as evidence that switching to SZC improves these clinical outcomes. Rather, they suggest that preservation of serum magnesium may represent an additional biological effect of selective potassium binding that deserves further investigation in appropriately designed prospective studies.

Another important finding is that the observed increase in serum magnesium appeared robust despite the absence of clinically relevant changes in factors known to influence magnesium homeostasis. During the study period, no meaningful changes occurred in proton pump inhibitor therapy, phosphate binder therapy, active vitamin D analogues, calcimimetics, magnesium-containing medications, or dialysate magnesium concentration. Therefore, although residual confounding cannot be completely excluded, the likelihood that the observed magnesium increase was primarily attributable to changes in concomitant treatment appears limited.

Because proton pump inhibitors are well recognized to reduce intestinal magnesium absorption [34,35], we additionally evaluated the influence of concomitant PPI therapy. As expected, serum magnesium concentrations were consistently lower in patients receiving PPIs than in those not receiving PPIs. Nevertheless, serum magnesium increased significantly following the transition to SZC in both subgroups. Although this subgroup analysis was limited by the relatively small sample size, these findings suggest that the association between switching to SZC and higher serum magnesium concentrations cannot be explained solely by concomitant PPI use.

The present study complements previous clinical investigations of SZC in maintenance hemodialysis patients. The DIALIZE trial demonstrated that SZC effectively maintained predialysis serum potassium concentrations within the target range in patients undergoing chronic hemodialysis [20], while the subsequent DIALIZE-Outcomes trial demonstrated sustained potassium control but did not show a significant reduction in the primary composite cardiovascular endpoint [22]. In contrast, the primary objective of the present study was not to evaluate potassium-lowering efficacy but to investigate changes in serum magnesium and other biochemical parameters following replacement of conventional cation-exchange resins with SZC in routine clinical practice. Accordingly, our findings should be regarded as complementary to previous studies and provide additional insight into the metabolic consequences of changing potassium-binding therapy.

Several strengths of the present study deserve mention. First, clinical, biochemical, and nutritional parameters were prospectively evaluated every two weeks over the 12-week observation period, allowing detailed assessment of temporal changes in serum magnesium and related laboratory variables. Second, subgroup, correlation, and longitudinal analyses consistently demonstrated that the increase in serum magnesium was observed irrespective of the previously prescribed resin and was not associated with prior resin dose or the magnitude of potassium change. Third, information regarding medications known to influence electrolyte and mineral metabolism was collected and incorporated into the analyses, thereby reducing the likelihood that the observed findings were explained by major changes in concomitant therapy. In addition, the dialysate magnesium concentration remained unchanged throughout the study period, minimizing another potential source of variation in serum magnesium concentrations [35].

This study also has several limitations. It was conducted at a single center with a relatively small sample size, and nearly half of the initially eligible patients were excluded from the final analysis. Although most exclusions resulted from institutional infection-control measures requiring isolation dialysis, hospitalization, or treatment modification rather than treatment-related adverse events, the possibility of selection bias cannot be completely excluded. Furthermore, the study lacked a concurrent control group and was not designed to evaluate magnesium balance, intestinal magnesium absorption, fecal magnesium excretion, vascular calcification, cardiovascular outcomes, fracture risk, or patient survival. Consequently, causal relationships between switching to SZC and preservation of serum magnesium concentrations cannot be established on the basis of the present findings alone. Therefore, the present findings should be interpreted as exploratory and require confirmation in larger prospective studies.

## 5. Conclusions

In this prospective, single-arm, before-and-after study conducted in routine clinical practice, switching maintenance hemodialysis patients from conventional cation-exchange resins to sodium zirconium cyclosilicate was associated with a significant increase in serum magnesium concentrations over a 12-week observation period. Although serum potassium increased modestly after the treatment transition, concentrations remained within the recommended range for patients receiving maintenance hemodialysis, and no clinically meaningful changes were observed in nutritional status or other measured biochemical parameters.

Subgroup, correlation, and longitudinal analyses consistently supported the robustness of the observed increase in serum magnesium. These findings are consistent with the greater cation selectivity of sodium zirconium cyclosilicate and suggest that replacement of conventional cation-exchange resins may contribute to preservation of serum magnesium concentrations.

Because this was a single-center study with a limited sample size and without direct assessment of magnesium balance or clinical outcomes, the biological mechanisms and clinical implications of these findings remain to be established. Further multicenter prospective studies evaluating magnesium metabolism together with clinically relevant outcomes are warranted to confirm these findings and clarify their clinical significance.

## Figures and Tables

**Table 1:** Patient characteristics.

Item	Category/Statistic	*n*
*n*		28
Age, years	73 (68–81)	
Sex	Male	10
	Female	18
Duration of dialysis, years	7.5 (3.0–12.8)	
Primary disease	Diabetic nephropathy	11
	Chronic glomerulonephritis	7
	Nephrosclerosis	5
	ADPKD	1
	Lupus nephritis	1
	Unknown cause	3
Dose of SPS, g/day (*n* = 20)	6.54 (4.20–9.81)	
Dose of CPS, g/day (*n* = 8)	25 (25–50)	

Data are presented as median (IQR). Abbreviations: ADPKD, autosomal dominant polycystic kidney disease; CPS, calcium polystyrene sulfonate; SPS, sodium polystyrene sulfonate.

**Table 2:** Patient status before and after switching from potassium-binding resins to SZC.

Item	Before	After	*p* Value
K level, mEq/L	5.01 (4.66–5.39)	5.26 (4.89–5.48)	0.031
Corrected Ca level, mg/dL	9.04 (8.88–9.49)	9.24 (8.93–9.45)	0.368
IP level, mg/dL	5.10 (4.33–5.42)	5.23 (4.57–5.74)	0.127
Mg level, mg/dL	2.46 (2.23–2.66)	2.66 (2.35–2.93)	<0.001
whole-PTH, pg/mL	72.6 (58.6–106.8)	72.5 (54.5–102.9)	0.767
GNRI	91.4 (86.7–93.3)	91.4 (86.1–94.6)	0.750
Dose of SZC, g/week	–	20	–

Data are presented as median (IQR). *p* values were calculated using the Wilcoxon signed-rank test. Abbreviations: Ca, calcium; IP, inorganic phosphorus; Mg, magnesium; GNRI, Geriatric Nutritional Risk Index; SZC, sodium zirconium cyclosilicate.

**Table 3:** Subgroup analysis stratified by proton pump inhibitor (PPI) use.

	K Before	K After	*p* Value	Mg Before	Mg After	*p* Value
PPI group (*n* = 15)	4.96 (4.74–5.34)	5.27 (5.10–5.43)	0.073	2.27 (2.07–2.42)	2.35 (2.15–2.80)	0.010
Non-PPI group (*n* = 13)	5.09 (4.64–5.23)	5.20 (4.83–5.48)	0.168	2.61 (2.52–2.84)	2.76 (2.66–3.08)	<0.001

Data are presented as median (IQR), mEq/L for K and mg/dL for Mg. *p* values were calculated using the Wilcoxon signed-rank test (within-group, before vs. after). Abbreviations: K, potassium; Mg, magnesium; PPI, proton pump inhibitor.

**Table 4:** Subgroup analysis by prior resin type (SPS vs. CPS).

	K Before	K After	*p* Value	Mg Before	Mg After	*p* Value
SPS group (*n* = 20)	5.01 (4.69–5.23)	5.27 (5.06–5.48)	0.015	2.41 (2.26–2.64)	2.65 (2.36–2.96)	<0.001
CPS group (*n* = 8)	5.02 (4.73–5.57)	5.10 (4.81–5.48)	0.844	2.52 (2.19–2.63)	2.66 (2.23–2.81)	0.023

Data are presented as median (IQR), mEq/L for K and mg/dL for Mg. *p* values were calculated using the Wilcoxon signed-rank test (within-group, before vs. after). The magnitude of the magnesium increase (ΔMg) did not differ significantly between the SPS and CPS groups (Mann–Whitney U test, *p* = 0.823). Abbreviations: CPS, calcium polystyrene sulfonate; K, potassium; Mg, magnesium; SPS, sodium polystyrene sulfonate.

## Data Availability

Data cannot be shared publicly due to ethical reasons. Data will be made available on request from the Sekishinkai Social Medical Corporation Foundation Saitama District Ethics Committee to researchers who meet the criteria for accessing the confidential data (contact via Naofumi Ikeda, naofumi-ikeda@saitama-sekishinkai.org).

## Supplementary Materials

The following supporting information can be downloaded at https://www.mdpi.com/article/10.3390/jcm15145406/s1. Figure S1: Patient enrollment, exclusion, and final analysis flowchart.

## Abbreviations

The following abbreviations are used in this manuscript:

Ca	Calcium
CKD	Chronic kidney disease
CPS	Calcium polystyrene sulfonate
ESKD	End-stage kidney disease
GNRI	Geriatric Nutritional Risk Index
HD	Hemodialysis
IP	Inorganic phosphorus
IQR	Interquartile range
Mg	Magnesium
PD	Peritoneal dialysis
PPI	Proton pump inhibitor
whole-PTH	Whole parathyroid hormone
RCT	Randomized controlled trial
SPS	Sodium polystyrene sulfonate
SZC	Sodium zirconium cyclosilicate
