# Supplementary material for: Switching from Cation-Exchange Resin to Sodium Zirconium Cyclosilicate Hydrate in Patients on Hemodialysis
Source: J Clin Med. 2026 Jul 10;15(14):5406. doi: 10.3390/jcm15145406 (PMC13412515; doi:10.3390/jcm15145406)
Supplement: Supplementary file 1 [file jcm-15-05406-s001.zip › jcm-4379795-supplementary.pdf]

Supplementary Materials: The following supporting information can be downloaded: **Figure S1.** Patient enrollment, exclusion, and final analysis flowchart.

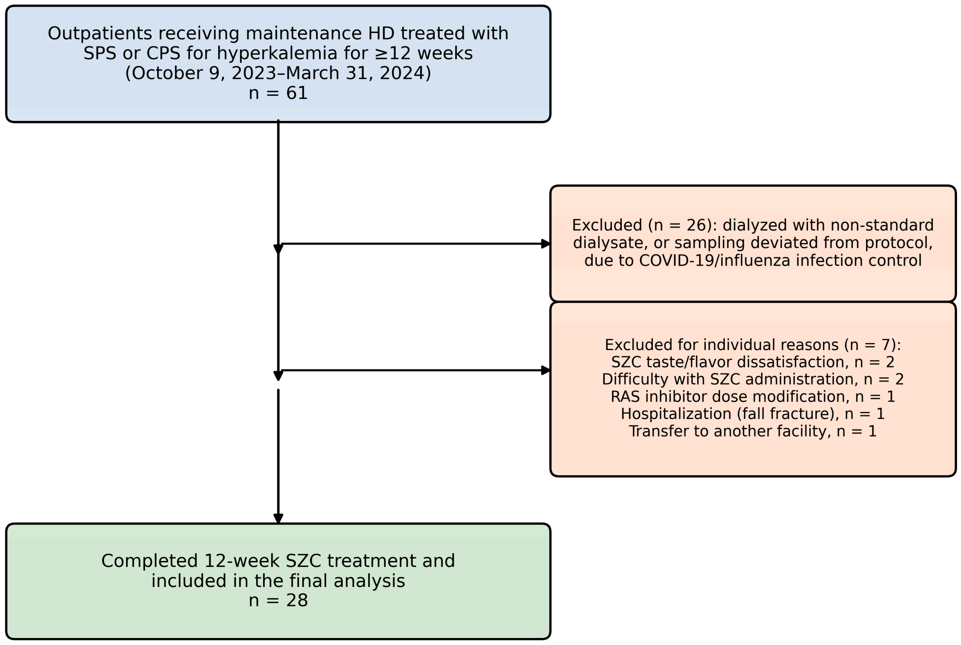

Figure S1.
